# Supplementary figures and images for: Prognostic factors of chronic pulmonary aspergillosis: A retrospective cohort of 264 patients from Japan
Source: PLoS One. 2021 Apr 1;16(4):e0249455. doi: 10.1371/journal.pone.0249455 (PMC8016288; doi:10.1371/journal.pone.0249455)

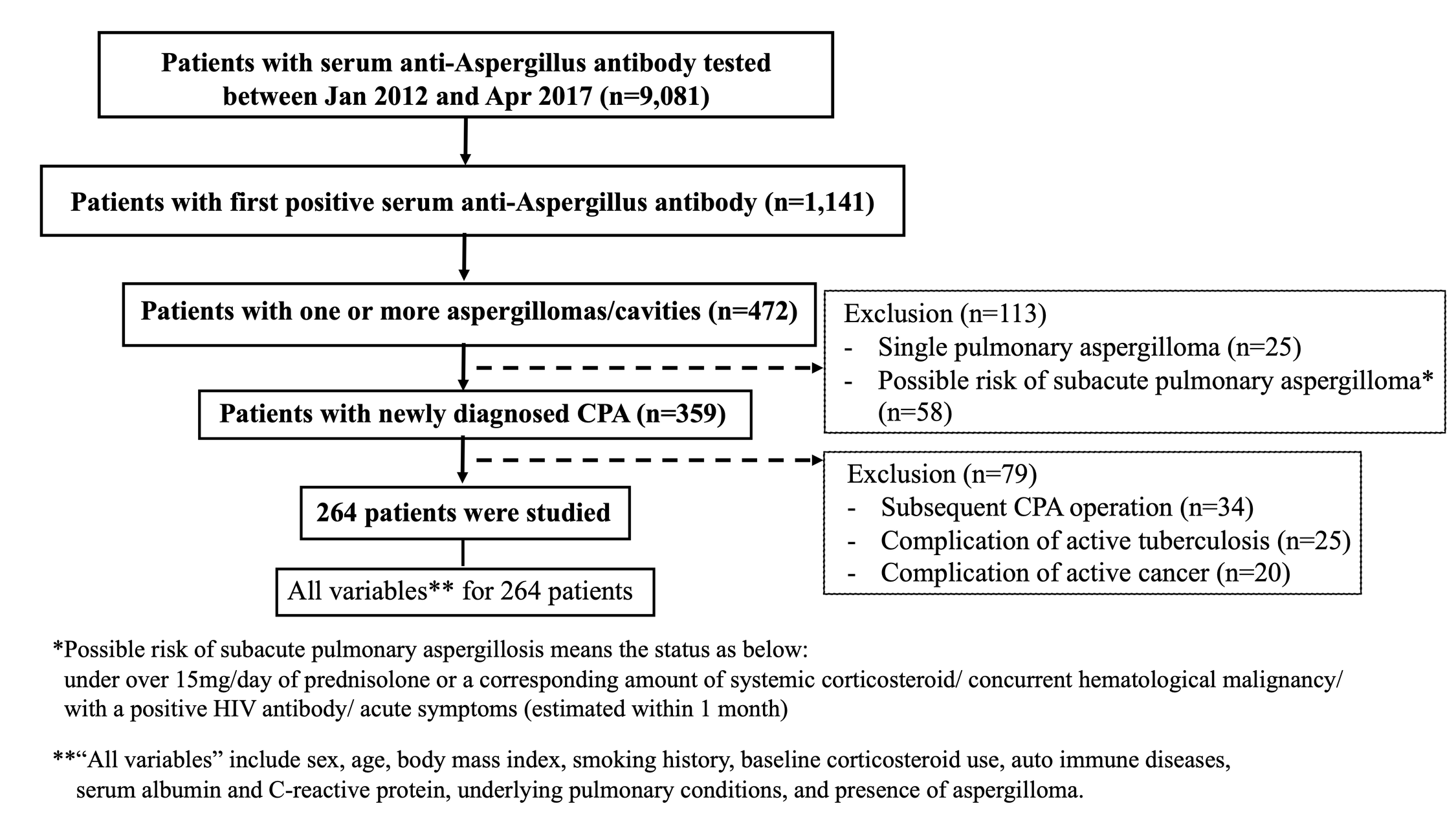

Supplement: S1 Fig — We screened patients with positive serum anti-Aspergillus antibody obtained between January 2012 and April 2017. After applying the exclusion criteria, we enrolled 264 newly-diagnosed chronic pulmonary aspergillosis patients. (TIF) [file pone.0249455.s001.tif]

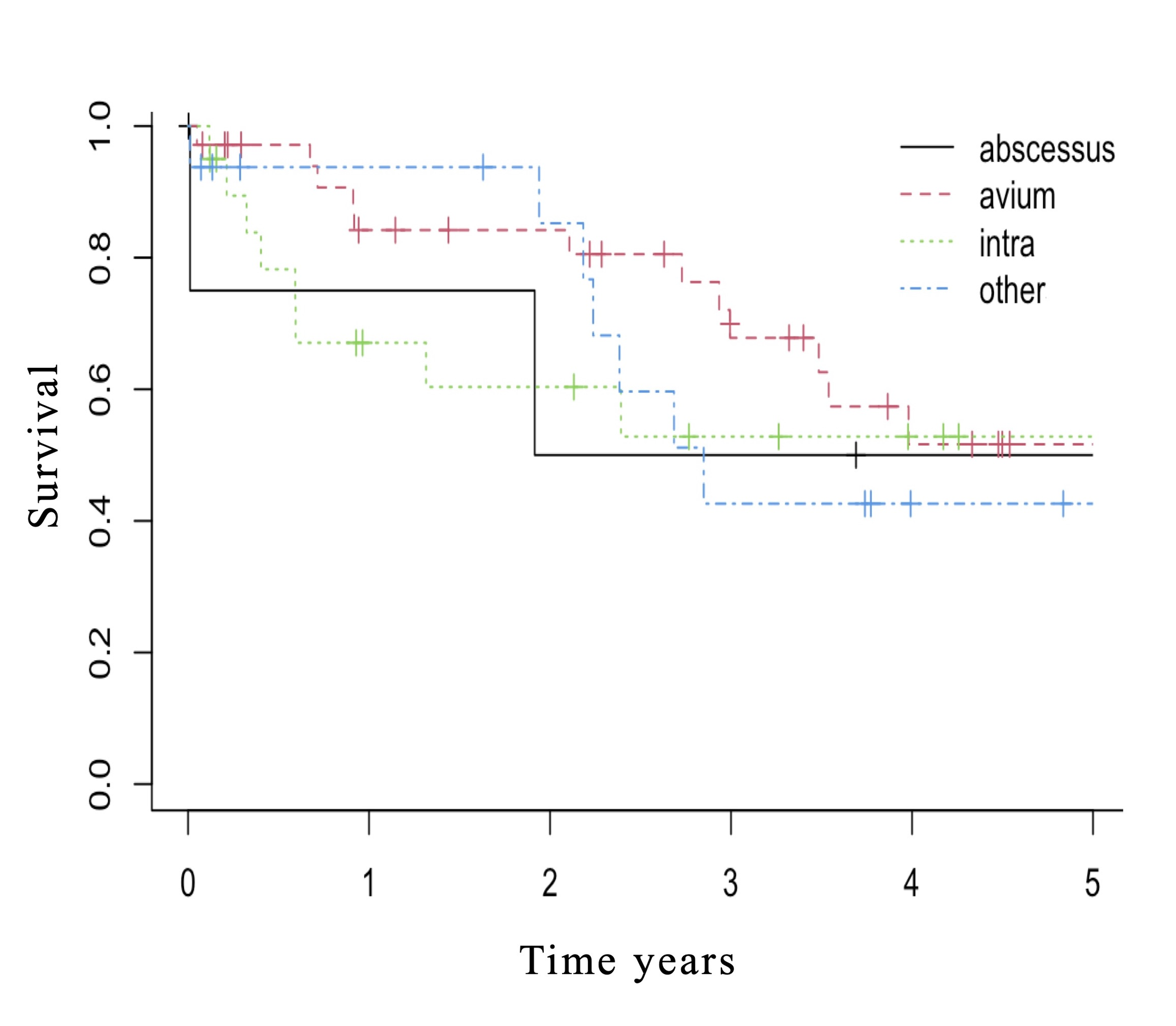

Supplement: S2 Fig — (TIF) [file pone.0249455.s002.tif]
