# Supplementary material for: Prognostic factors of chronic pulmonary aspergillosis: A retrospective cohort of 264 patients from Japan
Source: PLoS One. 2021 Apr 1;16(4):e0249455. doi: 10.1371/journal.pone.0249455 (PMC8016288; doi:10.1371/journal.pone.0249455)
Supplement: S1 Table — (DOCX) [file pone.0249455.s003.docx]

**S1 Table. Background characteristics grouped by anti-fungal drug administration.**

|  | administered (190) | non-administered (74) | p-value |
| --- | --- | --- | --- |
| Age, years | 70.3±10.9 | 72.7±11.5 | 0.11 |
| Male sex | 150 | 45 | <0.001 |
| Underlying pulmonary conditions |  |  |  |
| previous pulmonary tuberculosis | 121 | 35 | 0.004 |
| Non-tuberculous mycobacterium | 49 | 38 | <0.001 |
| Emphysema | 89 | 26 | 0.039 |
| Pneumothorax | 8 | 1 | 0.46 |
| Bronchiectasis | 18 | 28 | <0.001 |
| Thoracic surgery | 26 | 7 | 0.28 |
| Interstitial pneumonia | 37 | 16 | 0.86 |
| presence of aspergilloma | 73 | 15 | 0.002 |
| Albumin, g/dL | 3.2 [2.8-3.8] | 3.25 [2.8-4.0] | 0.46 |
