# Supplementary material for: Prognostic factors of chronic pulmonary aspergillosis: A retrospective cohort of 264 patients from Japan
Source: PLoS One. 2021 Apr 1;16(4):e0249455. doi: 10.1371/journal.pone.0249455 (PMC8016288; doi:10.1371/journal.pone.0249455)
Supplement: Attachment — Submitted filename: Review_Prognostic factors of CPA_retrospective chort of 264 patients from Japan.docx [file pone.0249455.s004.docx]

In their manuscript, the authors aim to identify factors that have an influence on mortality in a cohort of 264 CPA patients, focusing on underlying diseases as markers of prognosis.

The main finding of the work is the identification of interstitial lung disease / abnormalities (ILD/ILA) as a statistically significant prognostic marker. On the other hand, NTM-PD and other underlying diseases did not influence overall survival.

The cohort size is considerable; the results are interesting and deserve being reported.

However, a better definition of different CPA patterns and a more differentiated look at the underlying ILD are necessary.

**Major points:**

1. The different patterns of CPA in this cohort should be described.

Rationale: CPA is in itself heterogeneous, with its generally accepted patterns being Aspergilloma, Aspergillus nodule, CCPA, CFPA (not included here is SAIA, with overlapping features of IA).

These patterns probably describe a continuum of CPA disease, with CFPA, also sometimes referred to as „destroyed lung“, being the most severe, with extensive secondary fibrosis. Thus, when exploring prognostic factors in CPA, describing the CPA patterns is important.

2. The specific forms of the underlying ILD should be described.

Rationale: ILD is an umbrella term used to describe „diffuse parenchymal lung disease“, eventually leading to the development of pulmonary fibrosis. Etiology and prognosis are highly variable. Therefore, in the context of this paper, it is important to understand which forms of ILD were present in the CPA cohort. The type of ILD, such as Idiopathic pulmonary fibrosis (IPF), may determine an individual patient`s prognosis rather than the occurrence of CPA.

In this study, the radiological findings „honeycombing”, “upper lobe fibroelastosis” and “lower lobe fibrosis“, are used to define ILA. However, all of these changes also occur be secondary to CPA and therefore may present disease progression rather than ILD as an underlying disease.

The HRCT pattern of ILD should be described more specifically, (e.g. „UIP“, „NSIP“) and a clear definition of the underlying ILD should be attempted, such as:

- ILD with known etiology, such as ILD associated with systemic rheumatic disease
- ILD with unknown etiology such as Idiopathic interstitial pneumonia, e.g. IPF or Non-Specific Interstitial Pneumonia (NSIP)
- Others (e.g. ILD featuring cysts, such Pulmonary Langerhans cell histiocytosis or Pulmonary lymphangioleiomyomatosis)
- CPFE (Combined pulmonary fibrosis and emphysema)

**Minor points:**

In general, the discussion could be improved, incorporating the major points mentioned above. There are several minor points that should be addressed by the authors. Throughout the manuscript, the terms „complicating“ and „underlying“ seem to be used interchangeably, which is a bit misleading in some sentences. A professional language editing service may be considered to improve expression and clarity.

Abstract (page 2)

Line 27: Should read „we conducted a retrospective cohort study of…

Line 35: Should read „NTM-PD was not associated with higher mortality“, as this would be the logical hypothesis

Introduction:

Page 3:

Line 46: should read „Patients with CPA have a variety of underlying pulmonary conditions“, because CPA is usually the complication of other lung diseases and not vice versa

Line 50 ff.: This sentence merely repeats the statement of the previous sentences

Page 4:

Line 55: should read „were associated with ILD“

Line 56: should read „underlying NTM-PD“

Page 5:

Lines 75-77: should read „We had limited CPA cases to cases with a confirmed…“, otherwise the sentence gets the wrong meaning

Results:

Page 13

Lines 210, 215, Table 3: serum albumin and CrP: although it may be obvious, it should be mentioned that **low** albumin and **elevated** CrP (which cut-offs were used?) were prognostic markers, not just albumin and CrP

Discussion:

Page 16:

Line 264: should read „we found that underlying ILA was associated“

Page 17:

Line 274: the acronym „HOT” is used for the first time here and should be explained“

Line 278 ff.: As a possible explanation of these findings, it should be discussed that a subset of the CPA cases reported to complicate NTM-PD could be in fact aspergillus colonization.

Given the overlap in both symptoms and radiological appearance between NTM-PD and CPA, colonization and infection are particularly hard to differentiate.
