# Supplementary material for: Prognostic factors of chronic pulmonary aspergillosis: A retrospective cohort of 264 patients from Japan
Source: PLoS One. 2021 Apr 1;16(4):e0249455. doi: 10.1371/journal.pone.0249455 (PMC8016288; doi:10.1371/journal.pone.0249455)
Supplement: Attachment — Submitted filename: Response to Reviewers.docx [file pone.0249455.s005.docx]

Dear Editor and Reviewers

March 16^th^, 2021

Thank you very much for reviewing our manuscript and offering valuable advice.

We have addressed your comments with point-by-point responses, and revised the manuscript accordingly.

To facilitate your review of our revisions, the following is a point-by-point response to the questions and comments delivered in your letter dated March 11^th^, 2021.

REVIEWER #1 COMMENTS:

1. Do you assessed for Pneumocystis jirovecii coinfection?

- RESPONSE:
  No patient in this study had image findings typical of Pneumocystis pneumonia (PCP), so we did not perform bronchoalveolar lavage test for PCP. In some cases, the elevation of serum Beta-D-glucan was detected, but we think it was attributable to CPA.

2. What is your suggestion for prevention of CPA in patients with underling diseases?

- RESPONSE:
  Since *Aspergillus* species are common in our surrounding environment, it is difficult to completely prevent contacting with them. But it may be useful to avoid circumstances filled with mold or to wear a mask in such circumstances in order not to develop CPA.

3. What were your lab test for diagnosis of active cases of CPA? Only antibody?

- RESPONSE:
  We usually perform the following tests for assessing CPA: serum Beta-D-glucan and anti-*Aspergillus* antibody, bacteriological inspections (of sputum/ samples from bronchoscopy), and pathological examination (of sputum/ samples from bronchoscopy). We have limited CPA patients to those with confirmed positive serum anti-*Aspergillus* antibody to secure homogeneity.

REVIEWER #2 COMMENTS:

1. The different patterns of CPA in this cohort should be described.
   Rationale: CPA is in itself heterogeneous, with its generally accepted patterns being Aspergilloma, Aspergillus nodule, CCPA, CFPA (not included here is SAIA, with overlapping features of IA).
   These patterns probably describe a continuum of CPA disease, with CFPA, also sometimes referred to as „destroyed lung“, being the most severe, with extensive secondary fibrosis. Thus, when exploring prognostic factors in CPA, describing the CPA patterns is important.

- RESPONSE:
  As you mentioned, there are many patterns of *Aspergillus* infectious diseases: aspergilloma, *Aspergillus* nodule, SAIA, CCPA, and CFPA. In this study, aspergilloma and *Aspergillus* nodule cases were not included because of the inclusion criteria (We added some explanations for clarity in the manuscript). And we did not distinguish between CCPA and CFPA because it is actually difficult to do it, and because the distinction has not been made in most of recent CPA prognostic studies.

2. The specific forms of the underlying ILD should be described.
   Rationale: ILD is an umbrella term used to describe „diffuse parenchymal lung disease“, eventually leading to the development of pulmonary fibrosis. Etiology and prognosis are highly variable. Therefore, in the context of this paper, it is important to understand which forms of ILD were present in the CPA cohort. The type of ILD, such as Idiopathic pulmonary fibrosis (IPF), may determine an individual patient`s prognosis rather than the occurrence of CPA.
   In this study, the radiological findings „honeycombing”, “upper lobe fibroelastosis” and “lower lobe fibrosis“, are used to define ILA. However, all of these changes also occur be secondary to CPA and therefore may present disease progression rather than ILD as an underlying disease.
   The HRCT pattern of ILD should be described more specifically, (e.g. „UIP“, „NSIP“) and a clear definition of the underlying ILD should be attempted, such as:
   • ILD with known etiology, such as ILD associated with systemic rheumatic disease
   • ILD with unknown etiology such as Idiopathic interstitial pneumonia, e.g. IPF or Non-Specific Interstitial Pneumonia (NSIP)
   • Others (e.g. ILD featuring cysts, such Pulmonary Langerhans cell histiocytosis or Pulmonary lymphangioleiomyomatosis)
   • CPFE (Combined pulmonary fibrosis and emphysema)

- RESPONSE:
  Thank you for providing these insights. In consideration that few cases had undergone serum tests for assessing the type of ILA/ILD and pathological examination (of specimens from bronchoscopy/lung surgery), we believe a clear definition of the underlying ILD is difficult.
  Following your advice, we have assessed the HRCT patterns of ILA/ILD more precisely by making five categories: ①UIP/②NSIP/③PPFE pattern/④combined pulmonary fibrosis and emphysema/⑤unclassifiable. The breakdown of the categories was added into the RESULTS section. We have not performed survival analysis grouped by the categories because the sample size of each category is too small.

3. Minor points

- RESPONSE:
  We really appreciate your pointing out our unsuitable expressions in detail. We have dealt with them one by one. Some points we would like to give comments were as below.
  - Line 50 ff.: This sentence merely repeats the statement of the previous sentences

・RESPONSE: In this sentence, we want to clarify that most common underlying pulmonary conditions in CPA patients are COPD and tuberculosis.

- Results:
  Page 13
  Lines 210, 215, Table 3: serum albumin and CrP: although it may be obvious, it should be mentioned that low albumin and elevated CrP (which cut-offs were used?) were prognostic markers, not just albumin and CrP

・RESPONSE: We have corrected “serum albumin, C-reactive protein” to “lower serum albumin, elevated C-reactive protein”. The linearity of the relationships between the outcome and serum albumin/C-reactive protein was not problematic, so we did not adopt cut-offs for them.

Again, we appreciate all of your insightful comments. We worked hard to be responsive to them. Thank you for taking the time and energy to help us improve the paper.

Sincerely,

Yuya Kimura, M.D.

National Hospital Organization Tokyo National Hospital,

3-1-1 Takeoka, Kiyose-shi, Tokyo, 204-8585, Japan

Tel. +81-42-491-2111, Fax. +81-42-494-2168

E-mail. [yuk.close.to.wrd.34@gmail.com](mailto:yuk.close.to.wrd.34@gmail.com)
